# Global trends and hotspots in research for sudden hearing loss over the past two decades: a bibliometric and visualization analyses

**DOI:** 10.3389/fneur.2025.1561326

**Published:** 2025-05-20

**Authors:** Xueshi Di, Tiantian He, Yuqi Zhou, Xiaonan Li, Juanjuan Feng, Chenyang Su, Peng Bai

**Affiliations:** ^1^Department of Acupuncture and Moxibustion, The Third Affiliated Hospital of Beijing University of Chinese Medicine, Beijing, China; ^2^Department of Acupuncture and Moxibustion, Dongzhimen Hospital Affiliated to Beijing University of Chinese Medicine, Beijing, China; ^3^School of Acupuncture-Moxibustion and Tuina, Beijing University of Chinese Medicine, Beijing, China

**Keywords:** sudden hearing loss, bibliometric analysis, sudden sensorineural hearing loss, visual analysis, Citespace, VOSviewer

## Abstract

**Objective:**

This study aims to examine and visually map the characteristics, research hotspots, and emerging trends in sudden hearing loss research over the past two decades.

**Methods:**

A systematic search was conducted for English-language articles and reviews on sudden hearing loss published between 2004 and 2023 in the Web of Science Core Collection. Using Citespace, VOSviewer, and Bibliometrix, we performed a comprehensive analysis and visualization of publications, countries, institutions, authors, journals, and keywords.

**Results:**

The analysis identified 2,513 publications, 565 journals, 3,341 keywords, and 8,818 authors. The annual publication output has slightly increased. China has been the leading contributor to sudden hearing loss research, with strong collaborative ties to the United States. Hallym University ranks at the top among institutions. The journal Otology & Neurotology has the highest publication volume, while Laryngoscope is the most frequently co-cited journal. Key terms such as “deafness,” “sensorineural hearing loss,” and “therapy” dominate the keyword landscape. The pathogenesis and treatment of SHL are at the core of this research field, while “recovery,” “management,” and “prognostic factors” are gradually receiving broader attention.

**Conclusion:**

This study compiles and analyzes studies related to sudden hearing loss over the past 20 years, and presents bibliometric-based visual data on the progress and hotspots of sudden hearing loss research to provide researchers with references to help guide future sudden hearing loss research.

## Introduction

Sudden hearing loss (SHL) is defined as a rapid onset of hearing loss ≥30 dB in at least three consecutive frequencies within 72 h. SHL is a relatively rare condition, with an estimated incidence of 5–27 cases per 100,000 person-years, and approximately 66,000 new cases are reported annually in the United States (1). No significant difference in incidence has been observed between men and women. SHL typically affects one ear, although it can develop in both ears simultaneously (2). The pathogenesis of sudden sensorineural hearing loss (SSNHL), a subset of SHL, remains unclear. Potential etiologies include viral infections, disturbances in cochlear blood flow, autoimmune diseases, and endolymphatic hydrops. However, only 10–30% of cases have an identified cause, while the remaining cases are considered idiopathic (3–9). Some studies report hearing recovery rates ranging from 32 to 65% without active treatment, typically within 2 weeks of onset (10, 11). However, American guidelines for SHL suggest that these figures may be an overestimation (1). Without prompt diagnosis and intervention, SHL can lead to progressive hearing deterioration in the affected ear, potentially impacting the contralateral ear as well (10, 12). Therefore, active treatment remains necessary. Due to the unclear pathogenesis of SHL, its treatment is not standardized and varies widely among practitioners. Commonly used therapeutic approaches include glucocorticoids, vasodilators to enhance microcirculation, antiviral drugs, and hyperbaric oxygen therapy (13–15).

Bibliometrics, the field of quantitative analysis of scholarly works, employs statistical methods to identify patterns and connections within published literature (16). This approach allows for a broad assessment of research trends and facilitates the identification of emerging patterns within a field (17). Insights gleaned from bibliometric analysis can guide researchers’ strategic decisions, thereby enhancing the precision and impact of future studies. Applying bibliometrics to the academic literature on SHL can provide valuable insights into the current state of research in this area. This method goes beyond basic statistical analysis, revealing trends in research evolution and key areas of focus, which can inform future research directions and optimize resource allocation. Additionally, bibliometrics can map academic collaboration networks among researchers, institutions, and countries. Visualizing these networks not only highlights global knowledge distribution and academic exchange patterns, but also provides a macro perspective on the academic influence and knowledge dissemination pathways in SHL research. In this sense, bibliometric analysis serves as both a tool to assess the current state of SHL research and a guide for navigating future research pathways. To better understand the frontiers and hot topics in SHL research, we present this bibliometric analysis of scientific publications in this field from 2004 to 2023.

## Materials and methods

### Data sources and search strategies

The data for this bibliometric analysis were obtained from the Web of Science Core Collection (WoSCC) database. To ensure a comprehensive search, journals in related fields were also consulted. The search strategy aimed to capture literature on sudden deafness, covering the period from January 1, 2004, to December 31, 2023. The search formula was as follows: (((((TS = (Hearing Loss, Sudden)) OR TS = (Sudden Hearing Loss)) OR TS = (Deafness, Sudden)) OR TS = (Sudden Deafness)) OR TS = (Idiopathic Sudden Sensorineural Hearing Loss)) OR TS = (Sudden Sensorineural Hearing Loss). This strategy included various terms related to sudden deafness to ensure comprehensive coverage. The search was limited to articles and reviews published in English, with no additional language screening criteria applied. The search was completed on April 3, 2024. Two researchers, Chongyang Zhang and Ting Pan, then independently checked the articles for eligibility.

### Data collection and analysis

Bibliometric data exported from WoSCC included information on authors, titles, abstracts, keywords, and cited references, and were exported in plain text format. Impact factors (IFs) were obtained from the latest version of the Journal Citation Reports (JCR). To examine the relationship between academic production and geographic regions, we considered the impact of economic development, downloading gross domestic product (GDP) data for each region from the World Bank website (18).

Bibliometrix, an R-based tool, was used for the processing, analysis, and visualization of scientific literature data. We applied Bibliometrix to generate overview maps of the literature and keyword word clouds. VOSviewer (version 1.6.18) was used to construct and visualize bibliometric maps, analyzing relationships between authors, institutions, journals, and keywords. CiteSpace (version 3.6.1) was employed to identify keyword emergence, clustering, and to create visual representations of knowledge mapping.

## Results

### General data

**Figure 1:**
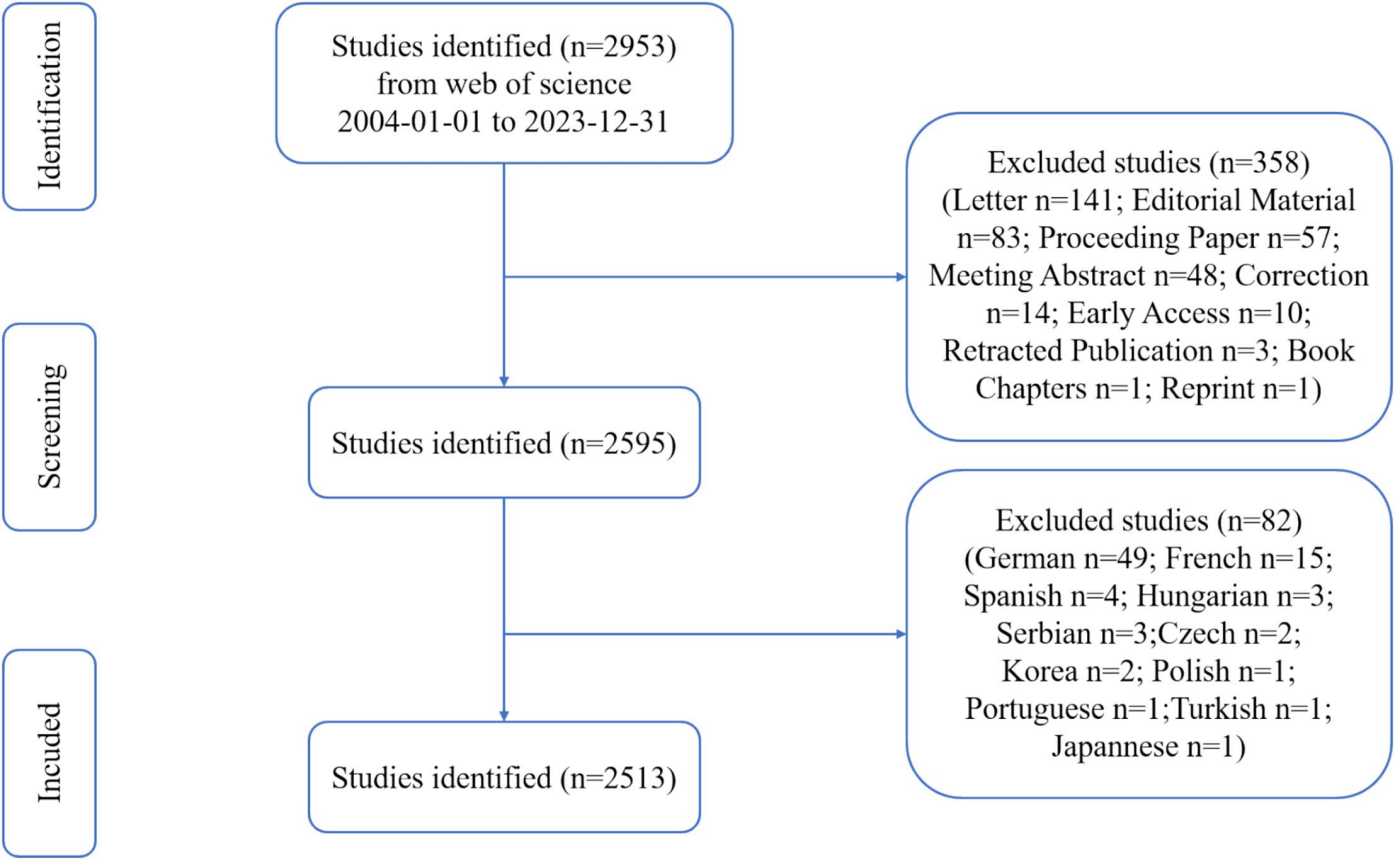
Flow diagram.

**Figure 2:**
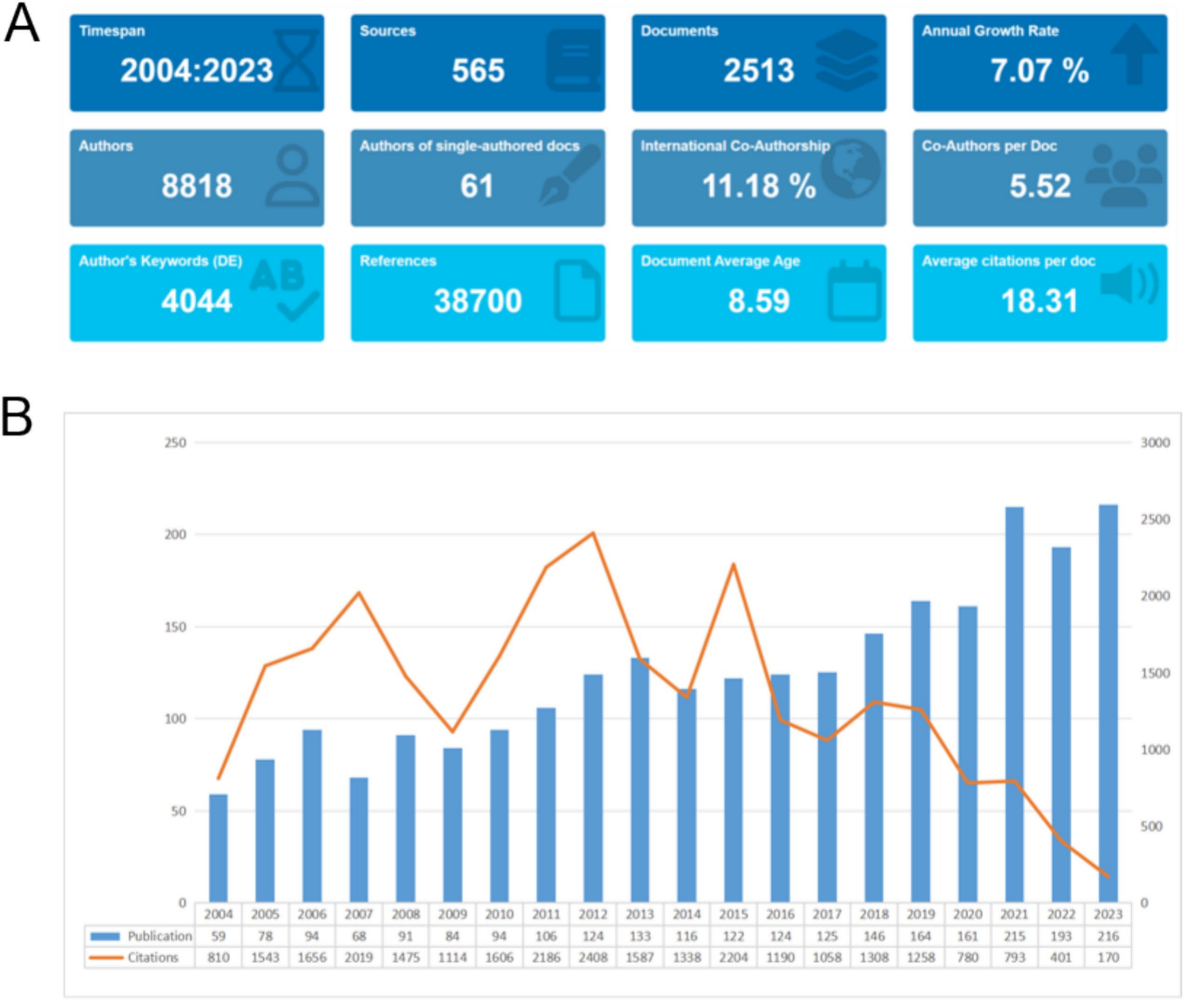
**(A)** General overview: the period of included articles, the number of journal categories, the total number of articles, the annual growth rate, the total number of authors, the number of articles published by a single author, the proportion of international co-authors, the number of co-authors of an article, the keywords given by the author, the number of references cited, the average life span of each article, the average number of citations per article. **(B)** Annual trends in publication volume and citation count: the figure shows the overall number of posts and citations in the SHL field from 2004 to 2023.

This study analyzed a total of 2,513 documents published between 2004 and 2023, with the data selection process depicted in Figure 1. As shown in Figure 2A, these publications were co-authored by 8,818 authors, reflecting a strong trend of academic collaboration, with international collaborations accounting for 11.18%. The average annual growth rate of publications was 7.07%, indicating rapid growth in the field during this period. The average number of citations per publication was 18.31, demonstrating a high academic impact. Figure 2B illustrates the annual distribution of publications and their citations. The number of publications increased significantly after 2020, likely due to a shift in research focus following the COVID-19 pandemic. First, there is emerging research indicating a potential link between COVID-19 and sudden sensorineural hearing loss (SSNHL) (19). This likely motivated many researchers to investigate this connection. Second, studies have shown that the COVID-19 pandemic, particularly the lockdown period, resulted in a significant increase in research productivity. For example, research found that in the 10 weeks following the lockdown in the United States, overall research productivity increased by 35% (20). Additionally, citation numbers peaked in years such as 2007 and 2012 but declined after 2020, possibly reflecting a shift in research focus or increased competition from emerging fields.

### Contributions of countries/regions to global publications

**Figure 3:**
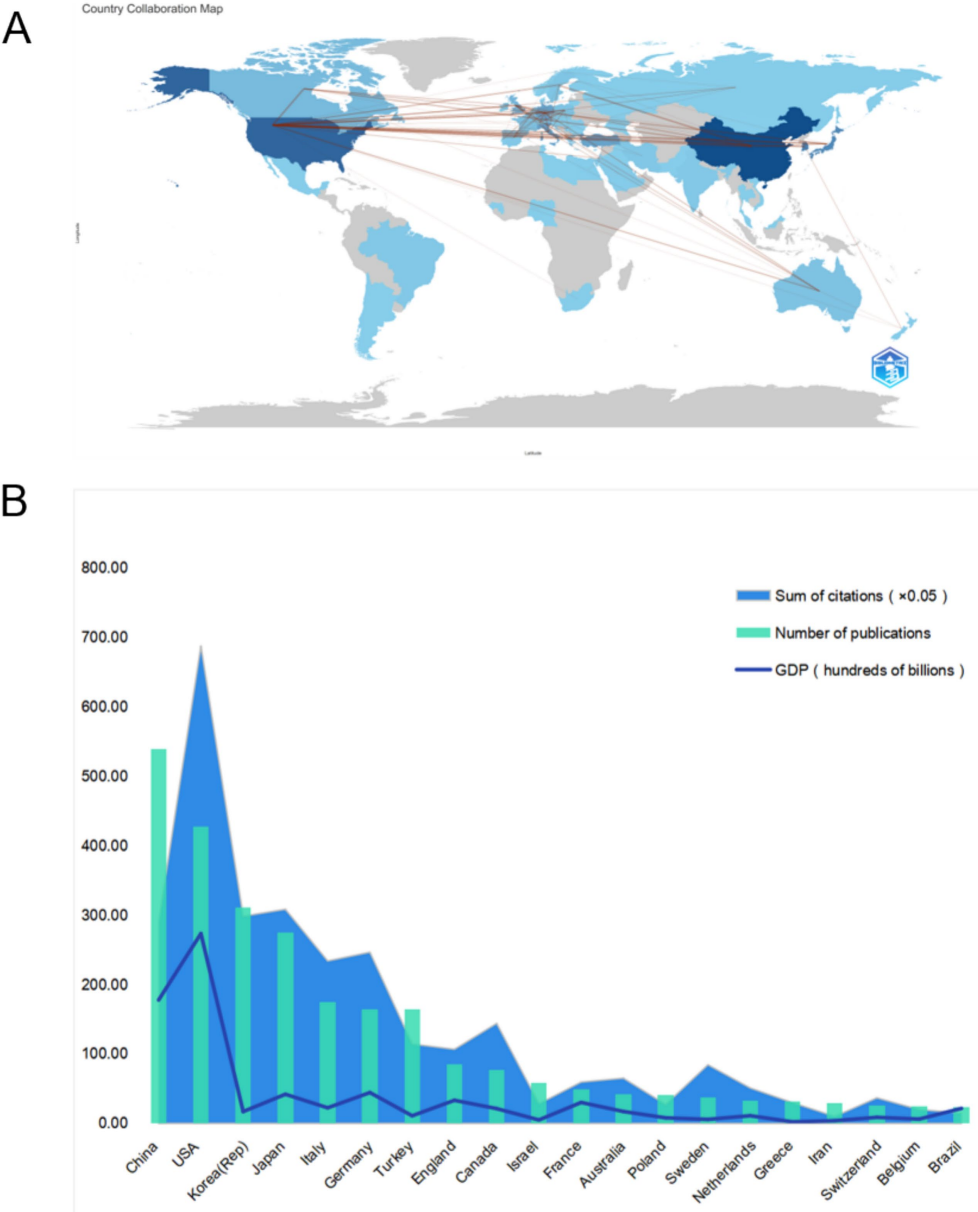
**(A)** Map of international collaborations: the lines connecting countries represent collaborative efforts, with thicker lines indicating stronger collaboration. The color intensity of each country indicates the total number of publications, with darker colors indicating more publications. **(B)** Countries in the top 20 international rankings in SHL studies: countries ranked in the top 20 in the world are evaluated according to the number of publications, the citation frequency and GDP.

International collaborations in the field of sudden hearing loss research are illustrated in Figure 3A. The United States and China are the core countries of the collaboration, with strong ties to European countries (e.g., Germany, the UK, and Italy) and East Asian countries (e.g., Japan and South Korea). Collaborations are mainly concentrated in North America, Europe, and East Asia, highlighting the scientific dominance of these regions and the strength of their international collaboration networks.

Figure 3B and Table 1 show the significant differences in the performance of the top 20 countries in terms of publications, citations, and GDP. China ranks first with 539 publications but has a relatively low number of citations. In contrast, the United States has slightly fewer publications (*n* = 428) but a much higher number of citations, reflecting its stronger research impact. South Korea and Japan are similar in terms of publications and citations. Despite its lower GDP, South Korea stands out for its high academic output and impact. Italy and Germany excel in both publications and citations, while the UK and Canada have fewer publications but higher citations, indicating an advantage in research quality. Overall, the relationship between publications, citations, and GDP is not strictly linear. The United States leads in citations due to its high-quality research, while China occupies an important position due to its large volume of published articles.

**Table 1:** Top 20 countries.

RANK	Country	Documents	Citations	GDP (hundreds of billions)
1	China	539	5,804	177.95
2	USA	428	13,761	273.61
3	Korea	311	5,974	17.13
4	Japan	275	6,172	43.13
5	Italy	175	4,684	22.55
6	Germany	165	4,936	44.56
7	Turkey	164	2,290	11.08
8	England	85	2,138	33.4
9	Canada	77	2,884	21.4
10	Israel	58	569	5.1
11	France	49	1,193	30.31
12	Australia	42	1,305	17.24
13	Poland	41	548	8.11
14	Sweden	37	1,691	5.93
15	Netherland	33	10,024	11.18
16	Greece	32	601	2.38
17	Iran	29	204	4.02
18	Switzerland	26	738	8.85
19	Belgium	25	401	6.32
20	Brazil	24	303	21.74

### Institutions with research publications on SHL

**Figure 4:**
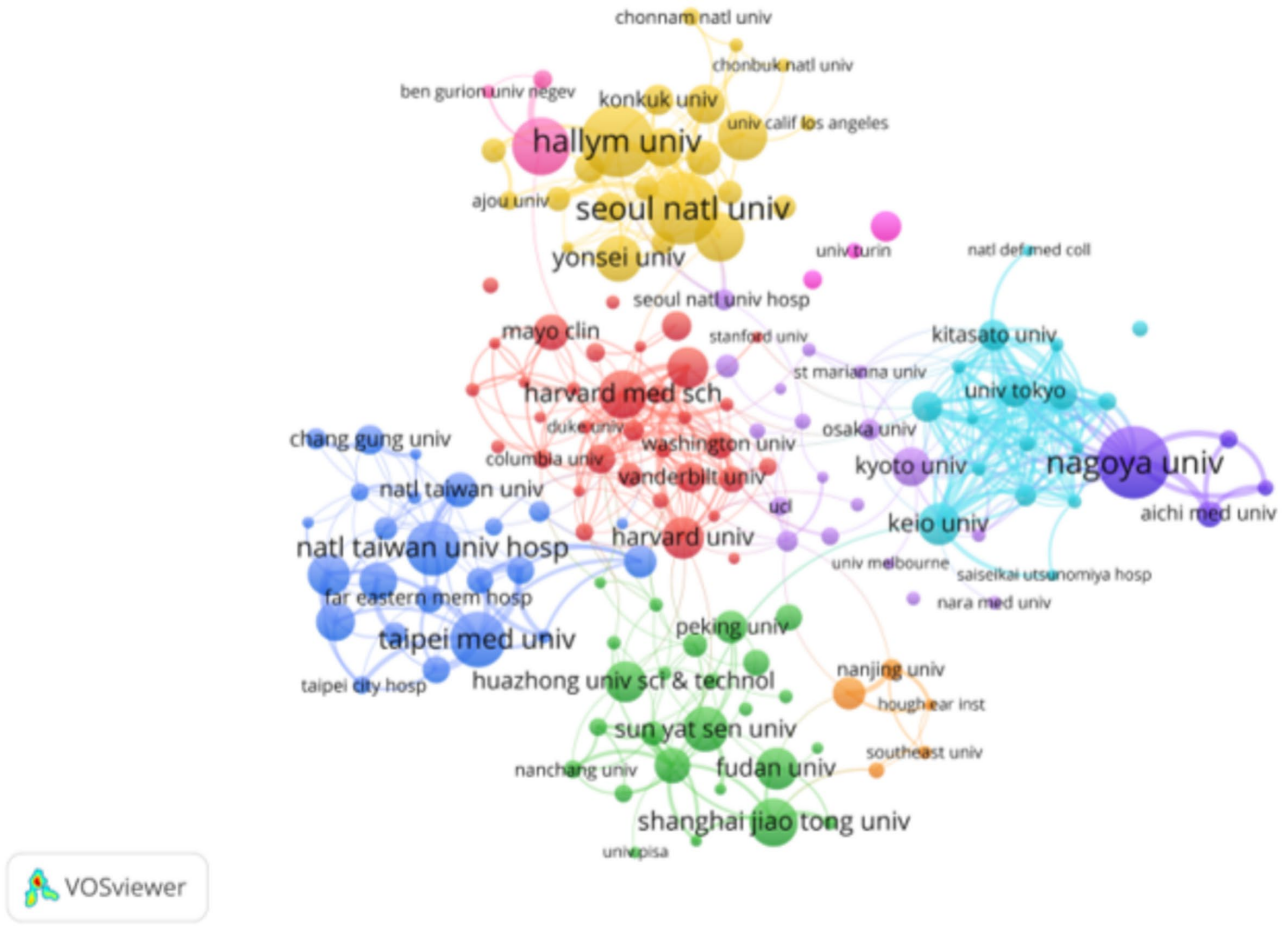
Institutional organization of the collaborative network: different colors represent different cooperative groups, the connection indicates cooperation, and the connection width indicates the close degree of cooperation.

Literature included in the study came from 2,480 institutions, and Table 2 presents the top 10 institutions with the highest number of publications. Among these, Hallym University, Nagoya University, and Seoul National University are central to the collaborative network, each publishing 44 articles and receiving a significant number of citations. Nagoya University stands out with 1,547 citations, as shown in the figure. Figure 4 illustrates the collaborative network of 187 institutions in the field of sudden hearing loss, each with at least 6 publications. Nagoya University forms a close collaborative group with other Japanese institutions, including the University of Tokyo and Kyoto University. Hallym University and Seoul National University maintain strong ties with other Korean universities, such as Yonsei University and Konkuk University. Harvard Medical School also demonstrates a high degree of collaboration with U.S. institutions and has a notable international impact, despite having a relatively low publication volume. Overall, the figure depicts the collaborative network of leading institutions in each country in the field of sudden hearing loss, highlighting both international research collaboration and the impact of regional research clusters.

**Table 2:** Top 10 institutions in terms of number of articles issued.

RANK	Institution	Documents	Citations
1	Hallym univ	44	704
2	Nagoya univ	44	1,547
3	Seoul natl univ	44	1,118
4	Tel aviv univ	34	264
5	Taipei med univ	33	839
6	Natl taiwan univ. hosp	32	531
7	Keimyung univ	29	948
8	Sungkyunkwan univ	29	1,333
9	Harvard med sch	28	321
10	Shanghai jiao tong univ	28	197

### Journals and co-cited journals

All publications in this study came from 565 journals, of which 68 journals contributed 5 or more publications. The references were sourced from 6,889 journals, with 178 journals receiving 50 or more citations. The top 10 journals in terms of publications and co-citations are shown in Table 3. Otology & Neurotology (*n* = 219) had the highest number of publications, accounting for 8.71% of all publications. Acta Oto-Laryngologica (*n* = 155) and European Archives of Oto-Rhino-Laryngology (*n* = 140) ranked second and third, respectively. The highest-ranked co-cited journal was Laryngoscope, which, despite having a relatively small number of publications, has a high number of citations, reflecting its quality and scholarly impact. The second and third most highly cited journals are Otology & Neurotology and Otolaryngology-Head and Neck Surgery, both of which are highly influential in the field of ear, nose, and throat research.

**Table 3:** Top 10 journals and co-cited journals.

Rank	Journal	Country	Documents	Citations	IF	Co-cited Journal	Co-citations	IF
1	Otology & neurotology	USA	219	5,154	1.9	Laryngoscope	4,856	2.2
2	Acta oto-laryngologica	Norway	155	2,552	1.2	Otology & neurotology	4,306	1.9
3	European archives of oto-rhino-laryngology	Germany	140	2,427	1.9	Otolaryngology-head and neck surgery	3,560	2.6
4	Journal of laryngology and otology	England	95	1,405	1.1	Acta oto-laryngologica	3,367	1.2
5	Laryngoscope	USA	94	3,845	2.2	European archives of oto-rhino-laryngology	2007	1.9
6	Otolaryngology-head and neck surgery	USA	79	3,153	2.6	Annals of otology rhinology and laryngology	1925	1.3
7	American journal of otolaryngology	USA	74	651	1.8	Hearing research	1,588	2.5
8	Journal of international advanced otology	Turkey	69	309	1	Jama otolaryngology-head & neck surgery	1,579	6
9	Audiology and neuro-otology	USA	59	1,286	1.6	Journal of laryngology and otology	1,244	1.1
10	Frontiers in neurology	Switzerland	59	298	2.7	Audiology and neuro-otology	1,136	1.6

**Figure 5:**
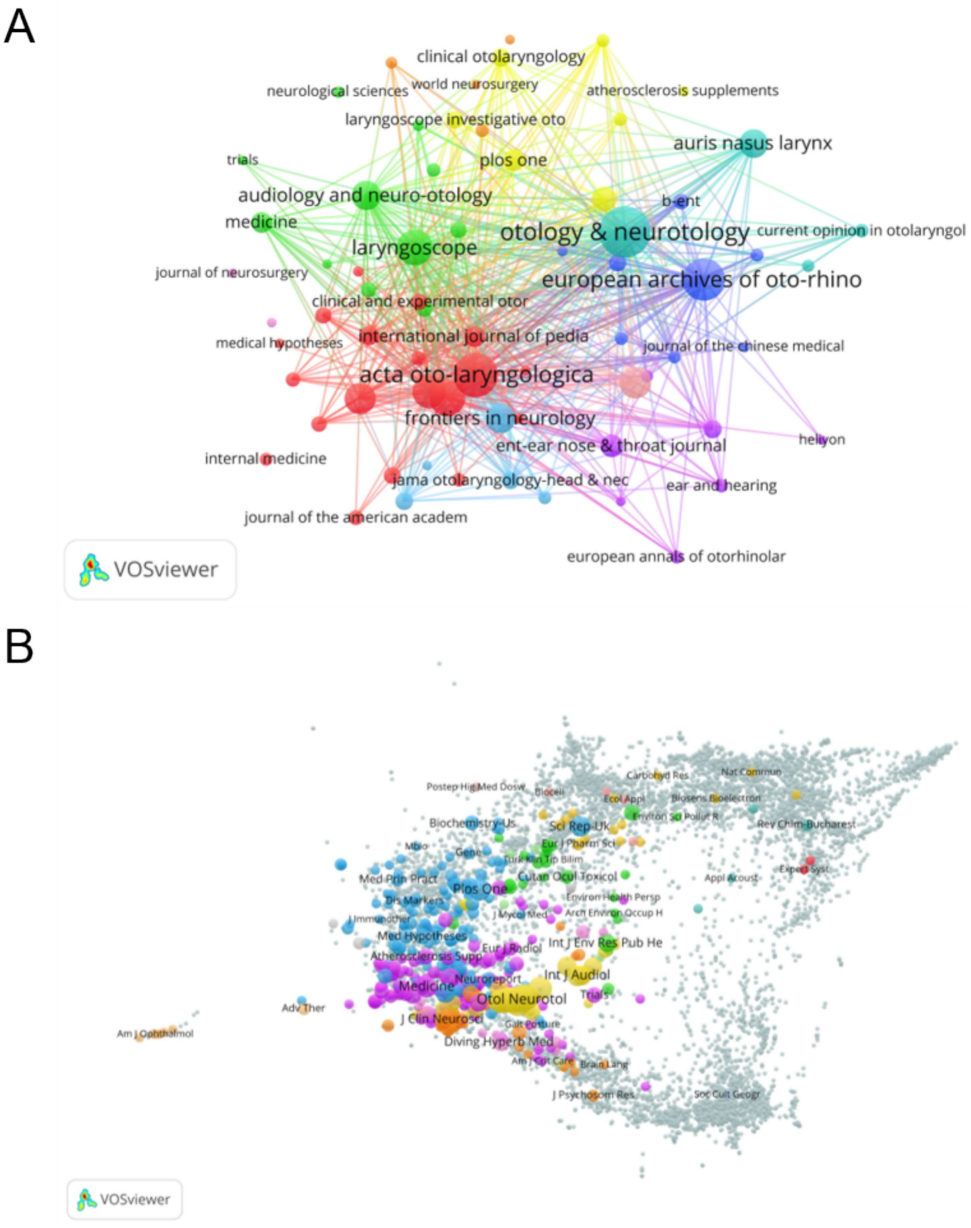
**(A)** Collaboration between the major journals: the size of the nodes reflects the influence of the journals, while the colors distinguish different Journals. The connecting lines between the nodes indicate the frequency of collaborations or cross-citations, with thicker lines indicating stronger collaboration. **(B)** Citation frequency distribution of different journal: the size of each node is proportional to the total number of citations received by the journal, with larger nodes indicating higher citation counts. The color of the nodes corresponds to different Journal.

Figure 5A illustrates the collaboration among major journals in the field of sudden hearing loss. The size of the nodes reflects the influence of the journals, while the colors distinguish different subject areas. The connecting lines between the nodes indicate the frequency of collaborations or cross-citations. The core journals Otolaryngology & Neurotology, Acta Oto-Laryngologica, and European Archives of Oto-Rhino-Laryngology are centrally positioned in the figure, demonstrating their importance in the field and their strong academic links. The dense collaborative network among these journals highlights the high relevance of the research topics and facilitates knowledge sharing and academic exchange. Figure 5B presents the citation frequency distribution of various journals in the field. The size of the nodes corresponds to the number of citations for each journal, with larger nodes, such as Otolaryngology & Neurotology and International Journal of Audiology, indicating their importance and high impact in the relevant research areas. The colors of the nodes represent different subject areas, highlighting interdisciplinary citation patterns. The strong connections between journals in the focus areas reflect the high citation frequency of these journals within similar or intersecting disciplines, underscoring their central role in advancing academic research and knowledge dissemination.

### Authors and co-citing authors

**Figure 6:**
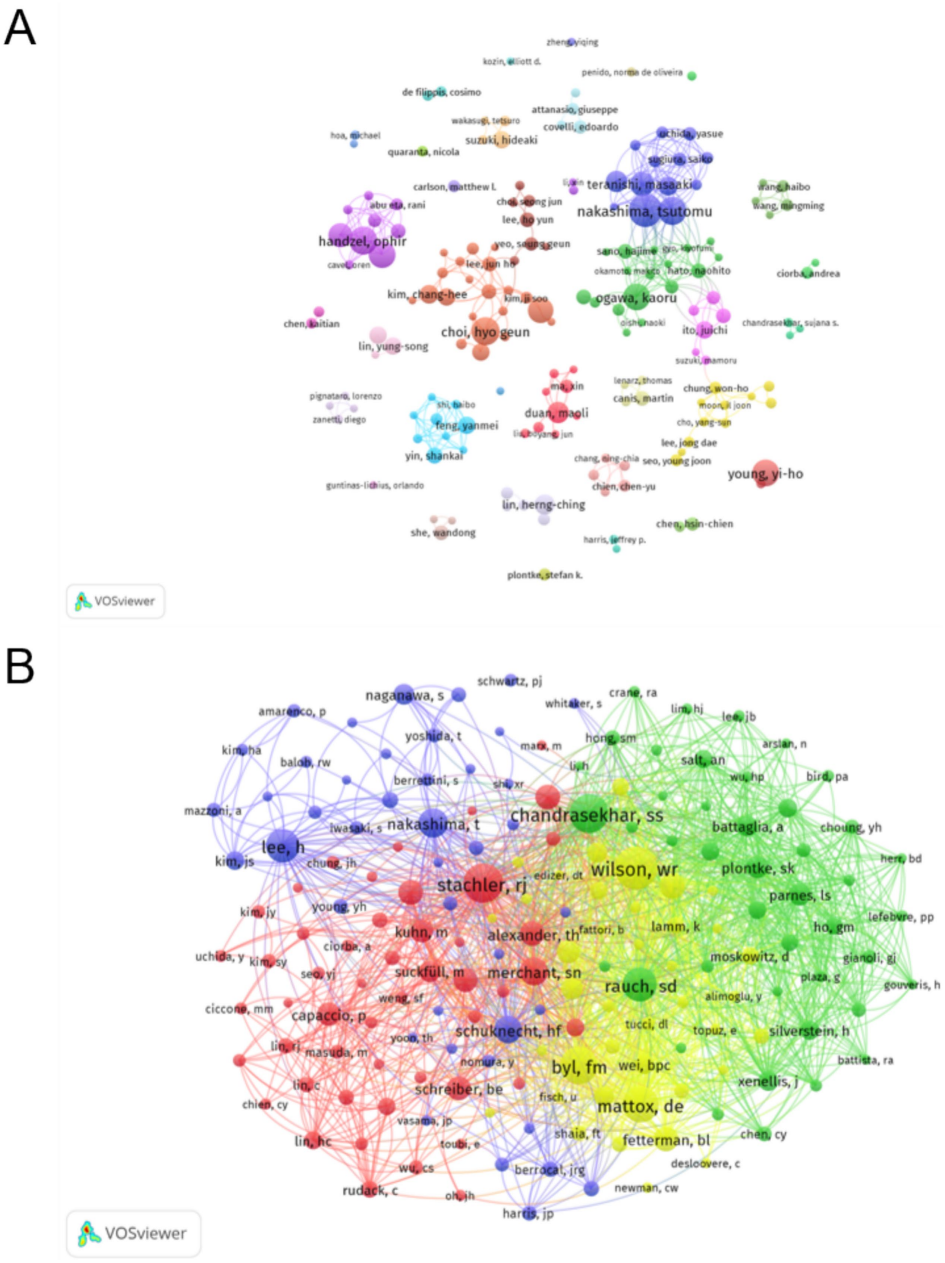
**(A)** Cooperative relationship among authors: different colors represent distinct research groups. The size of each node corresponds to the number of publications by each author, with larger nodes indicating higher publication output. The lines between nodes represent co-authorship, with thicker lines indicating stronger collaboration. **(B)** Cooperative relationship among co-cited authors: the node size represents the total number of citations of an author’s research, with larger nodes indicating more frequent citations by other studies. The thickness of the lines reflects the strength of the co-citation relationship between authors, with thicker lines indicating a stronger connection.

A total of 8,818 authors were involved in the study of sudden hearing loss. In Table 4, the most prolific author was Nakashima T, with 25 publications and 1,175 citations. Choi HG, Handzel O, and Sone M each ranked second with 21 publications. The top three authors in terms of citations were Wilson WR with 574 citations, Stachler RJ with 504 citations, and Chandrasekhar SS with 488 citations. Figure 6A depicts the collaborative network among authors in the field of sudden hearing loss research. Nakashima T is centrally positioned in the figure, forming a close-knit research team, demonstrating his central role in the field. Choi Hyo Geun and Handzel Ophir lead their respective collaborative groups, highlighting their influence in specific research areas. Other authors, such as Young Yi-Ho, Feng Yanmei, and Ogawa Kaoru, have also formed notable collaborative networks. Figure 6B shows the co-citation network of authors in the field of sudden hearing loss. Different colors represent various research groups, and Stachler RJ, Wilson WR, and Chandrasekhar SS have the highest number of co-citations, indicating their significant academic influence. In particular, Stachler RJ is at the center, reflecting the wide citation of his research and its role as a key reference for multiple research groups in the field.

**Table 4:** Top 10 authors and co-citing authors.

Rank	Author	Publications	Citations	Co-author	Citations
1	Nakashima T	25	1,175	Wilson WR	574
2	Choi HG	21	258	Stachler RJ	504
3	Handzel O	21	138	Chandrasekhar SS	488
4	Sone M	21	836	Byl FM	462
5	Ogawa K	20	425	Mattox DE	429
6	Ungar OJ	20	78	Rauch SD	384
7	Young YH	20	246	Lee H	371
8	Lee H	19	694	Nakashima T	293
9	Oron Y	18	72	Merchant SN	275
10	Teranishi M	18	1,027	Schuknecht HF	261

### Analysis of keywords in publications of SHL

**Figure 7:**
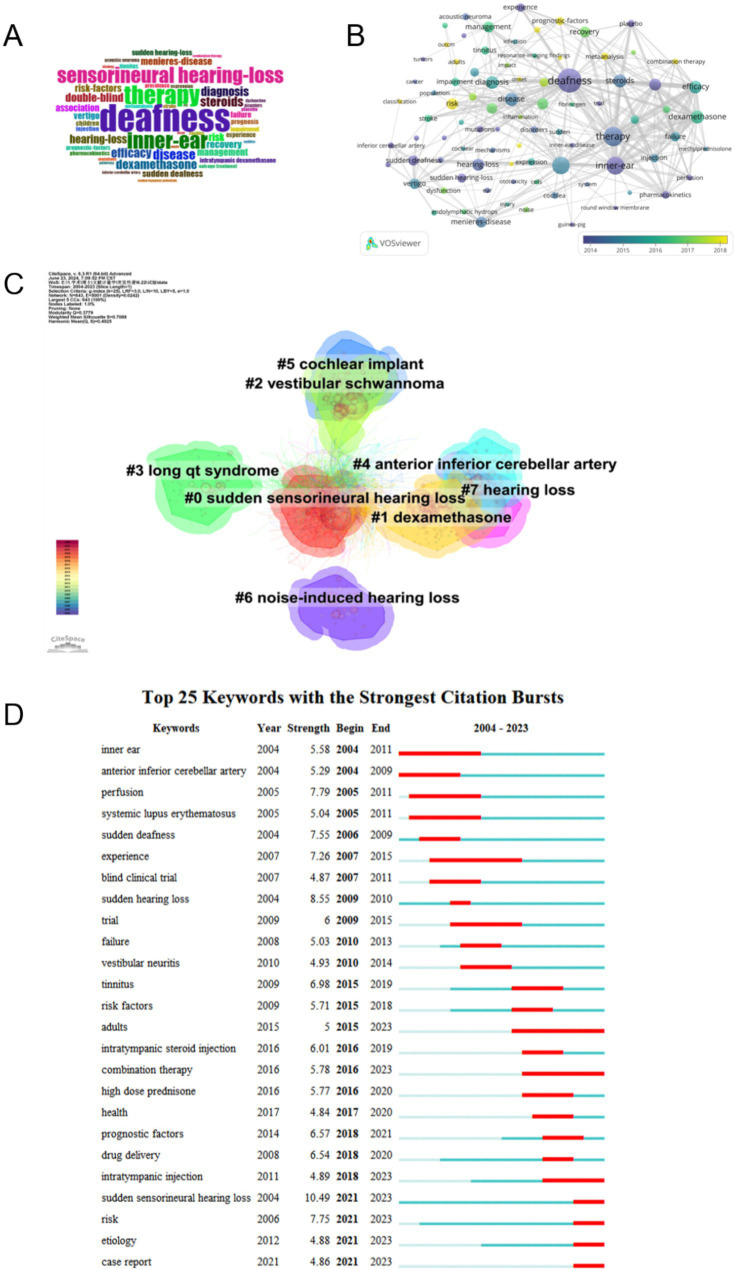
**(A)** Keyword word cloud: the size of each word corresponds to its frequency of occurrence, with larger words indicating higher frequency. **(B)** Overlay visualization of keyword frequency trends over time: keywords are color coded based on their average year of appearance. Purple represents earlier years, followed by blue for slightly later years, and green for subsequent years. Yellow indicates the most recent years. **(C)** Keyword clusters: the clustering analysis identifies eight research themes in SHL, each represented by a different color. **(D)** Top 25 Keywords with the strongest citation burst: the years between “Begin” and “End” represent the period when the keyword was more influential. Years in light green mean that the keyword has not yet appeared, years in dark green mean that the keyword is less influential, and years in red mean that the keyword is more influential.

In this study, 3,341 keywords were collected from 2,513 publications. Bibliometrix was applied to analyze the word cloud of keywords, as shown in Figure 7A. The size of the words in the word cloud represents the frequency of occurrence. The most prominent keywords included “deafness,” “sensorineural hearing loss,” and “therapy,” while other frequently appearing terms such as “inner ear,” “dexamethasone,” and “recovery” suggest that the disease mechanisms and treatments of sudden hearing loss are the main focus of research in the field.

We used VOSviewer software to analyze the keywords, identifying 86 terms with a frequency of 20 or more occurrences. A temporal trend visualization graph for these 86 keywords is shown in Figure 7B. The keywords are color-coded based on their average occurrence time in the 2,513 relevant publications, with blue terms representing earlier years and yellow and green terms indicating later years. Prior to 2016, the majority of studies focused on “therapy,” “deafness,” and “inner ear.” After 2016, “recovery,” “management,” and “prognostic factors” gained wider attention.

Keyword clustering analysis identifies the research hotspots and trends in the field. We used CiteSpace software to analyze keyword clusters with the results shown in Figure 7C. There are eight clusters each identified with different colors and marked with the main research themes including sudden sensorineural hearing loss dexamethasone vestibular schwannoma long QT syndrome anterior inferior cerebellar artery cochlear implant noise-induced hearing loss and hearing loss. We also performed an analysis of the emergence of sudden sensorineural deafness domain keywords as shown in Figure 7D. This figure presents the outbreak years intensity and start and end years for 25 relevant keywords between 2004 and 2023. Among these sudden sensorineural hearing loss emerged as the primary research hotspot in 2021 with the highest intensity (10.49). Sudden Deafness and Sudden Hearing Loss were the main research topics between 2004 and 2010 reflecting the concentration of research in the early years. Emerging keywords such as “risk,” “case report,” and “etiology” in recent years indicate a trend toward refinement and diversification of research in this field.

## Discussion

This study is the first to use bibliometrics to measure the development trend of SHL research from 2004 to 2023. Bibliometric visual maps are tools that represent and analyze data graphically, helping to reveal patterns, trends, and relationships. After excluding 530 studies that did not meet the inclusion criteria, 565 journals, 2,513 papers, and 38,700 cited references were eligible for analysis. To better understand SHL and identify emerging trends, bibliometric techniques and visual analysis tools were used to analyze national regions, journals, research institutions, authors, references, and keywords.

Bibliometric analysis revealed a slight annual increase in publications related to sudden hearing loss over the past 20 years. Several factors may explain this growth trend. Although the incidence rate of sudden hearing loss is low (approximately 5–27 cases per 100,000 people), its significant impact on patients’ daily lives results in substantial clinical and economic burdens, especially in countries with larger populations (12, 21). Additionally, the annual incidence of sudden hearing loss is increasing, and public awareness of health issues is gradually rising, which may lead to greater investment in research on this disease. Moreover, advancements in medical technology have introduced new diagnostic and treatment methods, sparking increased interest in SHL research.

Advanced diagnostic techniques, such as high-resolution three-dimensional Magnetic Resonance Imaging (MRI) and Magnetic Resonance Angiography (MRA), play a pivotal role in visualizing inner ear structures and evaluating blood flow—overcoming previous limitations in assessing the microcirculation of the human inner ear (22–25). In addition, genetic testing and proteomic analyses have significantly enhanced our understanding of the genetic and molecular mechanisms underlying SHL (26, 27). While corticosteroid therapy remains the mainstay treatment, there is currently no consensus on the optimal dosage and administration regimen. Meanwhile, emerging biologic therapies offer promising alternatives and new directions for clinical management (28, 29).

In terms of countries/regions, China has the most publications in the field of SHL, followed by the USA, South Korea, and Japan. The high publication volume from China can be attributed to its large research population and national investment in the field. With China’s rapid economic development and increasing public demand for healthcare, funding for the medical and health sectors has been steadily increasing. This may explain the rapid development of SHL research in China. As a powerhouse in scientific research, the United States also excels in SHL research, ranking second in terms of publication volume.

However, despite China’s high volume of publications, the citation rate of these papers is relatively low. The United States has the highest citation rate, followed by Japan, South Korea, and China. Five of the top 10 journals in terms of publication volume were established in the United States, indicating a high level of international influence and recognition of American research. The United States’ leading position in research investment supports the quality and impact of its research. The GDP ranking shows that the United States and China benefit from large economic scales, which likely influence their research output and citation rates in SHL. However, the relationship between GDP and citation rate is not linear, suggesting that factors such as research policy, education systems, and cultural values also play an important role. Additionally, American research is often characterized by innovation, rigor, and interdisciplinary collaboration, key factors contributing to its high citation rate. The lower citation rate of Chinese literature reflects limitations in international influence, recognition, research quality, and innovation. This may be linked to conservative research methods, limited research topics, language barriers, and insufficient international cooperation. To address this, China should improve its scientific research evaluation system, increase funding, cultivate an innovative academic culture, and strengthen international cooperation. Enhancing the quality of English papers and training scientific research talent will also be key to achieving high-quality SHL research output.

Researchers should focus on journals with a high volume of publications and citations to stay abreast of the latest advancements in SHL research. This strategy ensures access to the most relevant and impactful findings and helps identify appropriate journals for manuscript submission, facilitating the timely dissemination of research. And the analysis of academic journals also reveals critical pathways for advancing SHL research. Otology & Neurotology (219 publications) demonstrate established leadership through their publication volume and citation metrics. Although Laryngoscope and Otolaryngology-Head and Neck Surgery do not publish as many articles, their high citation counts and total link strength suggest that the research they publish is highly recognized and influential in academia. The emergence of Audiology and Neuro-Otology (ranked 9th) and Frontiers in Neurology (ranked 10th) highlight an intersection between SHL and neurology, where interdisciplinary collaboration could benefit SHL studies.

Research has shown that during the acute phase of SHL, white matter damage occurs in the auditory nerve pathway, such as the medial geniculate body, with the extent of damage positively correlating with the severity of hearing loss (30). Following the onset of SHL, dynamic changes in brain functional network connectivity and neurovascular coupling are observed. Early stages are characterized by functional changes, while later stages involve structural alterations. These changes are closely linked to the duration and prognosis of the disease (31). SHL can also serve as an early indicator of central nervous system disorders, such as multiple sclerosis (MS), in which approximately 25% of patients experience sensorineural hearing loss (32). This is often accompanied by white matter lesions and abnormal auditory brainstem responses, suggesting that central nervous system inflammation contributes to auditory dysfunction. Thus, the pathology of SHL is closely tied to neural mechanisms, including dysfunction of the central auditory pathway, impaired neural plasticity regulation, and neuroinflammatory processes. These mechanisms not only influence hearing recovery but also significantly impact the development of neurological disorders. However, among the top 20 journals in SHL publication volume, all have an Impact Factor (IF) below 10. This may be due to two main factors: first, SHL research is a specialized field, which limits the breadth of citations and affects the journal’s IF; and second, SHL research may not involve a wide range of interdisciplinary fields, restricting the audience for its findings.

Four scholars whose articles have been cited more than 450 times are Wilson WR, Stachler RJ, Chandrasekhar SS, and Byl FM. Wilson’s high citation rate suggests that his contributions to SHL are likely seminal or foundational. Wilson discovered the relationship between viral seroconversions and SHL, showing that the incidence of viral seroconversions is greater among SHL patients, which suggests that viral infection is a major cause of SHL (7). Wilson also documented the effective role of corticosteroid therapy in managing SHL, marking a significant advancement in the treatment of this otological emergency (33). Moreover, Wilson presented a model to predict recovery from idiopathic sudden hearing loss (34). Nakashima, with the highest volume of publications, also plays a prominent role in SHL research. These scholars are key contributors, disseminating pivotal discoveries that significantly advance SHL research. By recognizing the distinct research domains of various authors, researchers can quickly identify potential collaborators, facilitating the timely generation of high-caliber scholarly articles.

Co-occurring keyword analysis serves as a pivotal method for identifying thematic trends and patterns within the literature on SHL (35). The findings delineates four pivotal research domains in sudden hearing loss (SHL): clinical trials, inner ear pathologies, cardiovascular risk factors, and therapeutic strategies. The prominence of clinical trials is attributed to three principal factors: (1) the idiopathic nature of SHL necessitates rigorous investigations to elucidate pathophysiological mechanisms; (2) patient heterogeneity and variable disease presentations demand randomized controlled trials (RCTs) for evidence-based protocols; and (3) the urgency to prevent irreversible hearing loss drives rapid clinical translation. Additionally, comparative efficacy assessments of corticosteroids, antiviral agents, and intratympanic therapies rely on methodologically robust trials to ensure reproducibility and regulatory compliance.

Inner ear pathologies involve multifactorial mechanisms, including hair cell dysfunction, spiral ganglion neuron degeneration, microcirculatory disruptions, and endolymphatic homeostasis imbalance (36–39). These processes may act independently or synergistically to induce auditory deficits. Notably, conditions like vestibular schwannoma and Ménière’s disease exacerbate SHL via elevated inner ear pressure and endolymphatic accumulation (40, 41). Therefore, a thorough investigation of the link between inner ear diseases and SHL is essential for advancing our understanding, improving diagnostic accuracy, and refining therapeutic approaches for SHL.

The growing correlation between cardiovascular risk factors and SHL is increasingly recognized, with evidence suggesting that systemic conditions can profoundly affect auditory functionality (42, 43). The inner ear’s blood supply is primarily maintained by the labyrinthine artery, the sole vascular conduit to this region. Cardiovascular risk factors can cause vascular constriction, arteriosclerosis, or other anomalies, impairing blood flow to the inner ear and potentially triggering or worsening SHL.

Therapeutic advancements emphasize systemic-local synergy. Systemic corticosteroids remain foundational for their anti-inflammatory and immunomodulatory effects (1, 44), while intratympanic administration achieves localized drug delivery, enhancing cochlear bioavailability and minimizing systemic toxicity (45–49). Current research prioritizes optimizing dosing regimens, formulations, and pharmacokinetic profiles to maximize therapeutic windows. For refractory cases, cochlear implants with advanced signal-processing algorithms demonstrate improved speech perception, marking a paradigm shift in rehabilitative strategies (50).

Burst keywords highlight emerging research hotspots. These keywords gain significant attention over time and are frequently cited in research articles. In this study, we identified 25 relevant terms with the strongest citation bursts, which likely indicate the research hotspots of their respective time periods. The keyword burst analysis shows that SSNHL research can be divided into three distinct phases.

Between 2004 and 2009, a surge in keywords like sudden deafness and anterior inferior cerebellar artery (AICA) reflects an increased focus on SHL’s pathophysiological mechanisms and associated vascular pathologies. AICA syndrome, in particular, may have seen significant development during this period. From 2010 to 2015, the terms intratympanic steroid injection and trial emerged as focal points, marking a shift toward exploring therapeutic modalities and conducting clinical trials. The inclusion of terms such as experience and blind clinical trial underscores the emphasis on clinical practice and rigorous scientific methodology. Between 2016 and 2023, keywords like combination therapy, high-dose prednisone, and drug delivery gained prominence, suggesting a move toward more complex and personalized treatment strategies. Prior to 2016, research on SHL primarily focused on therapeutic interventions, deafness, and the inner ear. This emphasis reflected an urgent need to understand the underlying mechanisms of hearing loss, identify effective emergency treatments, and explore the physiological basis of deafness within the inner ear. The primary goal was to provide immediate intervention to prevent permanent hearing loss and address the underlying pathology of the inner ear whenever possible. After 2016, the focus of SHL research broadened to encompass recovery, management, and prognostic factors. This shift represents a maturation in the field, transitioning from acute intervention to a more comprehensive, long-term approach. Researchers have found that approximately two-thirds of patients experience spontaneous improvement, with maximum recovery typically occurring within 2 weeks. This evolution in research led to increased interest in identifying the factors that influence recovery, such as the degree of hearing loss, audiogram patterns, age, and the presence of vertigo. Additionally, attention shifted to understanding which patients are most likely to recover, as well as the timing and extent of recovery. The changing focus of key terms highlights the growing interdisciplinary nature of SHL research. The mention of systemic lupus erythematosus (SLE) and risk factors signals growing interest in the connection between SHL and systemic diseases, which is crucial for prevention and early intervention. Furthermore, the keywords adults and health suggest a focus on the impact of SHL on adult health and quality of life. Terms like intratympanic injection and drug delivery highlight innovations in treatment administration techniques. As interdisciplinary research expands and methodologies evolve, SHL studies are advancing toward more precise and personalized treatment strategies. Future research is likely to concentrate on personalized treatments, molecular-level disease mechanisms, and improving patient quality of life.

## Limitations

There are some limitations to this study. First, to ensure high-quality bibliometric analysis, this study relied on journals with an impact factor identified in the Web of Science database, which is the most renowned database of scientific publications across various research topics. However, it is possible that some landmark studies on sudden hearing loss with high citation counts may have been excluded from the search because they were published in non-SCI journals or other databases, potentially affecting the results of the study. Second, in order to maintain consistency in the analysis and avoid potential complications arising from language barriers, we filtered studies published in English, which may mean non-English writing papers were underestimated. Third, VOSviewer and CiteSpace do not offer advanced statistical analysis functions, which may introduce statistical bias. Therefore, these tools cannot fully replace systematic retrieval. Additionally, this research started in April 2024. Given that it aims to explore a full span of 20 natural years, the literature published in 2024 was not incorporated. Although these limitations may slightly affect the overall results, they are unlikely to alter the global trends in SHL presented in this paper.
